# Serum Immunoglobulin Levels and Complement Function of Tannery Workers in Bangladesh

**DOI:** 10.5696/2156-9614-9.21.190308

**Published:** 2019-03-14

**Authors:** Laila N. Islam, Fahimur Rahman, Aktar Hossain

**Affiliations:** Department of Biochemistry and Molecular Biology, University of Dhaka, Dhaka, Bangladesh

**Keywords:** Bangladesh, tannery workers, IgG, IgA, IgE, C3, C4, bactericidal activity, chromium

## Abstract

**Background.:**

Occupational exposures to chromium (Cr), which can have adverse effects on immune function, have not yet been extensively investigated. Hexavalent chromium (Cr (VI)), used in mineral tanning processes, poses a threat to the health of workers in the leather tanning industry.

**Objectives.:**

The aim of the present study was to evaluate the effects of long-term Cr exposure on the physical health and immunological parameters of male tannery workers in Bangladesh compared with a control group.

**Methods.:**

A health examination was performed with tannery workers (N=195) and control subjects (N=125) by physicians, demographic data were recorded in questionnaires and peripheral blood samples were collected. Serum Cr levels were analyzed by an atomic absorption spectrophotometer (AAS), immunoglobulin (Ig)G, IgA, and complement components C3 and C4 were determined by nephelometry, IgE was measured by sandwich enzyme-linked immunosorbent assay and complement function was assayed by bactericidal activity.

**Results.:**

The mean duration of work exposure for the tanners was 9.4±7.1 years. Their body mass index (21.8±3.0 kg/m^2^), was not significantly different from the controls (22.7±3.2 kg/m^2^). The mean serum level of Cr in 30 long-term exposed tannery workers (26.97±21.11 μg/dL) was significantly higher than that of 30 randomly selected control subjects (7.38±6.81 μg/dL). The tannery workers had rough skin, rashes, itchy and decolorized skin, allergic diseases and respiratory illness, and had significantly lower levels of serum IgG, IgA, C3 and C4, but significantly higher levels of IgE than the controls. IgG, IgA and C3 levels were all inversely associated with Cr, while IgG, IgE and bactericidal activity showed an inverse correlation with duration of exposure.

**Conclusions.:**

The results of the present study suggest that chronic exposure to Cr is associated with impaired immune function in male tannery workers.

**Participant Consent.:**

Obtained

**Ethics Approval.:**

The present study was approved by the Ethical Review Committee of the faculty of Biological Sciences, University of Dhaka, Bangladesh.

**Competing Interests.:**

The authors declare no competing financial interests.

## Introduction

Chromium (Cr) is an essential nutrient required for normal energy metabolism. Insufficient dietary Cr has been linked to maturity-onset diabetes and cardiovascular diseases. However, Cr exposure can be hazardous to human health. Among its many valence states, hexavalent chromium (Cr (VI)) is a major health concern. Epidemiological studies have reported a high incidence of lung cancer among workers occupationally exposed to Cr (VI) by inhalation.^1^,^2^ Ingestion of Cr (VI) compounds causes decreased hemoglobin content and hematocrit, increased total white blood cell and reticulocyte counts and plasma hemoglobin, which are indicative of intravascular hemolysis.^3^ Industrial exposure to Cr affects blood homeostasis by significantly increasing blood Cr concentrations, and long-term exposure can affect the immune system.^4^ Contact dermatitis has been found to be more prevalent in Cr-allergic patients exposed to leather products, which has become a serious concern in Denmark.^5^ Tannery workers in Bangladesh have been found to suffer from various allergic diseases.^6^ Occupational contact dermatitis has been reported in the leather workers in Indonesian tanneries.^7^

Tanning processes involve several chemicals and some of them are known to be potentially carcinogenic with adverse health effects. The tanning process involves basic trivalent chromium (Cr(III)) sulfate binding to collagen fibers which stabilizes the leather. Tannery workers are under constant risk of adverse health effects due to excessive exposure to nitrosamines, chromate pigments, soft sodium sulphide, lime, ammonium sulphate, benzidine-based direct dyestuffs, formaldehyde, leather dust and aromatic organic solvents.^8^ Chromium may enter the body via inhalation, ingestion and/or by direct contact. Tannery workers are exposed to Cr, mainly in the inorganic Cr (III) form or protein-bound form, such as leather dust.^7^ Cr (VI) is highly reactive and can penetrate the cell membrane and become reduced to Cr (III) upon binding to cellular proteins; the resulting conjugate may then function as a contact allergen. In the leather tanning industry, allergic contact dermatitis is common due to Cr (VI) exposure.^9^ Keratinocytes found in the skin are the first cells affected by Cr and can cause contact dermatitis.

Several studies have investigated the possible effects of Cr on lymphocytes. A study in Guiyu, China, found DNA (deoxyribonucleic acid) damage in lymphocytes in the umbilical cord blood of neonates.^10^ In a study on phytohaemagglutinin-induced blastogenesis in human lymphocytes, Cr (VI) showed a stimulatory effect at the lowest concentrations tested and an inhibitory effect at higher concentrations.^11^ Similar observations were reported in other studies, in which exposure to concentrations of Cr (VI) (10 and 100 μM) significantly decreased cell viability and increased apoptosis in both resting and activated lymphocytes.^12^ In an study on murine lymphocytes, the proliferation of both T and B lymphocytes and the production of immunoglobulin by lipopolysaccharide-stimulated B cells were significantly inhibited by cobalt-chromium particles after intraperitoneal injection in mice, which indicate immunosuppression by direct exposure to these heavy metals.^13^

**Abbreviations**
*BMI*: Body mass index
*C*: Complement component
*cfu*: Colony forming units
*ELISA*: Enzyme-linked immunosorbent assay
*Ig*: Immunoglobulin
*IL*: Interleukin
*PBS*: Phosphate buffered saline

A study investigating metal-induced modulation of nitric oxide production by murine macrophages in vitro found that Cr, copper and lead moderately suppressed inducible nitric oxide synthase while other heavy metals like cadmium, mercury, or cobalt did not produce any significant effect. These observations suggest that environmental toxicant metals may directly modify the nitric oxide synthase enzyme or cofactor activity and have suppressive effects on phagocytic function of macrophages to exercise defense mechanisms.^14^ Chromium exposure has a regulatory response on cytokine production and release. One study found mitogen-stimulated bovine peripheral blood mononuclear cells showed decreased interleukin (IL)-2, interferon gamma and tumor necrosis factor-α production.^15^ Another study showed that chromium niacinate decreased IL-6, IL-8 and monocyte chemoattractant protein-1 secretion and oxidative stress in U937 monocytes. Chromium niacinate supplementation also lowered blood levels of pro-inflammatory cytokines tumor necrosis factor α and IL-6.^16^ Exposure to manual metal arc stainless steel-welding fumes, which contain Cr and nickel, is immunosuppressive in mice and suggests a correlation between welding fume exposure and adverse respiratory health effects in humans.^17^

Immunoglobulin (Ig)G is the principal immunoglobulin of adaptive immune response that gives protection against invading pathogens and their toxins, while IgE functions against intestinal helminths and IgA gives mucosal immunity. Complement components C3 and C4 play important roles in bacterial killing, inflammation and cellular activation. These immunological parameters may be affected by chronic exposures to chemicals used in mineral tanning processes. Previous studies evaluated serum IgE levels and investigated their relationship with a wide range of allergic diseases, including allergic contact dermatitis, allergic rhinitis, allergic urticaria and non-specific allergy in tannery workers.^18^ In light of the difficulties in obtaining immunological data for tannery workers, the present study was undertaken to evaluate the humoral or soluble immune function in Cr-exposed tannery workers in Bangladesh.

## Methods

Tannery workers were enrolled in the present study in Hazaribagh, the largest industrial tanning area in Bangladesh, located in the southwestern part of the capital city of Dhaka. This cross-sectional study was conducted with 320 subjects: 195 tannery workers exposed to Cr and 125 unexposed control subjects. Inclusion criteria included at least two years of work in tanneries in sections with exposures to Cr. As there were very few female workers who met the inclusion criteria of two years of tannery work with Cr exposure, and in order to make equal comparisons, all of the control subjects were male.

The control subjects generally worked in the same region, in offices, shops, banks and student dormitories. Exclusion criteria included those suffering from impaired renal and liver functions and any other chronic conditions. Simple random and availability sampling was applied to collect samples.

The present study was approved by the Ethical Review Committee of the faculty of Biological Sciences, University of Dhaka, Bangladesh. Each individual was informed about the objectives and significance of the study. Only full consenting volunteers were enrolled in the study. The guidelines of the Ethical Review Committee were followed during physical health examination, participant interviews and peripheral blood sample collection.

### Data collection

Demographic information, including age, body weight, height, blood pressure, working hours per day, duration of work exposure (years) and type of work etc., was recorded by questionnaire (*Supplemental Material*). The health of participants was assessed by a physician.

### Blood sample collection

This study was conducted from July 2013 to June 2015. About 6 mL of peripheral blood was collected from each participant and was allowed to clot, and serum was separated by centrifugation. Serum samples were collected from the supernatant in small aliquots in Eppendorf tubes and stored at −20°C until analysis.

### Health examination classification

During sample collection, an expert physician/dermatologist examined the physical health of study participants. Rough and decolorized skin was clearly visible. Allergic urticaria was characterized by hives or swollen skin with rash, notable for its pale red, raised, itchy bumps. Allergic contact dermatitis was characterized by eczematous skin lesions usually observed after contact with certain chemicals. Itchy skin from unknown causes was classified as a non-specific allergy. Allergic rhinitis was characterized by watery exudation of the conjunctivae and nasal mucosa as well as sneezing and coughing. A food allergy was an immediate hypersensitivity reaction involving itching, swelling in the throat, and difficulty in breathing, and sometimes included gastrointestinal problems (diarrhea, gas formation, and cramping), rashes, and headaches caused by ingestion of certain foods. Scaly, superficial infections on skin and nails by fungus and bacteria were also noted. The health information of the study subjects was carefully recorded on questionnaires which were subsequently analyzed for demographic data.

### Chromium analysis

Samples were analyzed for the measurement of Cr by an atomic absorption spectrophotometer (AAS) (Varian 240) that used acetylene flame to vaporize the acid-treated samples. The AAS utilized a hollow cathode lamp specific for Cr detection in gaseous state and detected the total Cr content in the samples. Only 30 serum samples from the workers involved in activities in various sections of the tanneries (at least 3 from each section) and samples of 30 randomly picked control subjects were investigated for this parameter due to a scarcity of serum, as one milliliter of each sample was necessary for this analysis.

### Reagents

All reagents used for the determination of immunoglobulins IgG and IgA, and complement components C3 and C4 were purchased from Siemens Healthcare Diagnostics, GmbH, Germany (https://www.healthcare.siemens.com).

### Serum analysis

IgG was measured using the BN ProSpec^®^ system for nephelometry. Upon reaction with a specific antibody, IgG contained in serum formed an immune complex that scattered a beam of light through the sample. The intensity of the scattered light was proportional to the concentration of IgG present in the sample. Serum IgA and complement proteins C3 and C4 were measured using the same device, following reaction with the corresponding specific antibody. The rest of the procedure was similar to that described for IgG.

The quantitative test for IgE was based on a solid phase enzyme-linked immunosorbent assay (ELISA) technique. The ELISA plate and the reagents were obtained from DRG Diagnostics, Germany. The sandwich ELISA technique utilized one anti-IgE antibody in the solid phase and another one in the antibody-enzyme conjugate solution. The test sample was added to the anti-IgE coated microtiter plates and the content of IgE in the serum samples was determined by standard procedure.

### Assay of complement function

Serum complement function was assessed by bactericidal activity on *Escherichia coli* DH5α cells, according to the procedure described elsewhere.^19^,^20^ Briefly, bacterial cells were grown in nutrient broth for 14–16 hours at 37°C. Then the cells were harvested, washed twice with phosphate buffered saline (PBS) and adjusted to 0.600 optical density at 620 nm. Aliquots of 200 μL of the bacterial cell suspension were mixed with 20 μL of serum samples and incubated for 30 minutes at 37°C. During incubation, serum complements exhibited bactericidal activity on *E. coli* cells, and the remaining viable cells were serially diluted with PBS to 1:10 000 and then 20 μL of each was spread on 3 agar plates, incubated for 16–18 hours, and the number of colonies formed was counted. For the negative control, 20 μL of PBS was added to the bacterial cell suspension, incubated and then serially diluted with PBS to 1:50 000 and spread on agar plates. The number of colonies formed was recorded.

### Statistical analyses

Data were analyzed using the Statistical Package for Social Sciences (version 17.0 for Windows, SPSS Inc., Chicago, USA). Student's t-test and nonparametric tests, including chi-squared and Mann-Whitney U tests (as appropriate) were used for comparison of different parameters between two groups (tannery workers and unexposed group). Spearman's rho bivariate tests were used for correlation analyses between different parameters of the study populations. GraphPad Prism 7.05 (Graphpad Software Inc., La Jolla, CA, USA) was used to analyze bactericidal activity data. The results were considered significant when p was ≤ 0.05.

## Results

Demographic data on the study subjects were collected in questionnaires and the findings were analyzed and presented in Table 1. The age of the tannery workers varied from 15 to 65 years with a duration of occupational exposure ranging from 2 to 38 years. The body mass index (BMI) of the tannery workers varied from 15.8 to 28.8 kg/m^2^ and their working hours ranged from 7 to 16 hours per day, including overtime. The age of the control subjects varied from 17 to 62 years and their BMI ranged from 16.2 to 31.5 kg/m^2^. About 15% of the tannery workers were underweight (BMI <18.5 kg/m^2^) compared to only 3% of the control group (p<0.001, χ^2^ = 15.18).

**Table 1:** Demographic Characteristics of Tannery Workers and Control Subjects

Parameters tested^*^	Tannery workers N=195	Control subjects N=125	Statistics p-value
Age (years)	32.1 ± 11.0	30.3 ± 8.9	NS
Body mass index (kg/m^2^)	21.8 ± 3.0	22.7 ± 3.2	NS
Systolic blood pressure (mmHg)	121.6 ± 9.0	118.7±12.4	NS
Diastolic blood pressure (mmHg)	78.1 ± 5.5	79.5±5.6	NS
Duration of work exposure (years)	9.4 ± 7.1	-	NA
Hours of work (per day)	10.4 ± 2.3	7.6±2.2	<0.05
Physical health examination data from questionnaire, total number (%)^#^			
(i) Rough skin, itch and rash	88 (45.1%)	3 (2.4%)	<0.001
(ii) Decolorization of skin	32 (16.4%)	-	N.A.
(iii) Food and other allergy	22 (11.3%)	5 (4.0%)	<0.05
(iv) Respiratory problems: rhinitis, cough, chest sound	24 (12.3%)	5 (4.0%)	<0.05
(v) Fungal and bacterial infection on forearm, leg and toes	27 (13.9%)	2(1.6%)	<0.01
(vi) Pain in different parts of the body	29 (14.9%)	4 (3.2%)	<0.01
(vii) General weakness	16 (8.2%)	4 (3.2%)	N.S.

^*^Student's t-test;

^#^Chi squared test.

Study participants were interviewed and examined at a temporary health check-up came set up at the workplace.

All values expressed in Mean ± SD. Abbreviations: NS, not significant; NA, not applicable.

**Figure 1:**
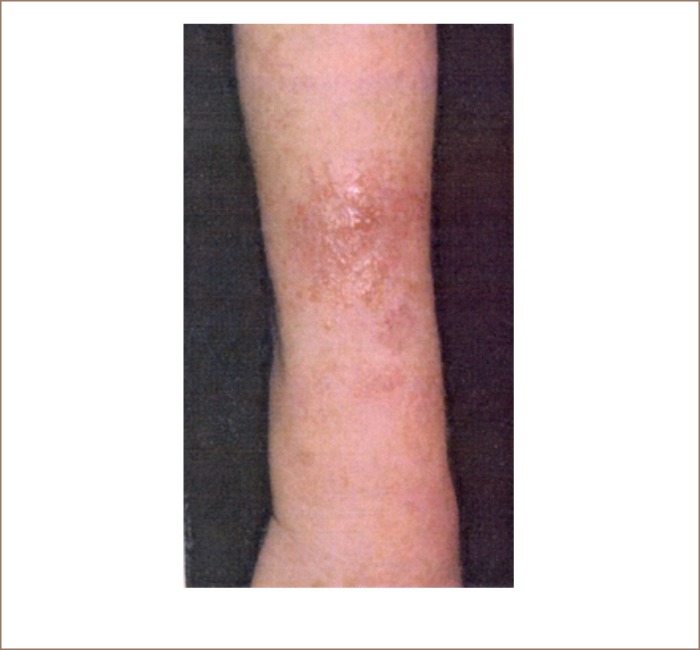
Skin manifestations (allergic urticaria) in tannery workers. Image used with permission.

The physical health examination data of the study subjects are also shown in Table 1. Of the tannery workers, 45.1% had rough skin along with contact dermatitis (itching and rashes) on both limbs, 16.4% had decolorized skin, 11.3% reported food and non-specific allergy, while 12.3% had respiratory problems and 13.9% were suffering from fungal and bacterial infections on body surfaces (*Figure 1*). All of these health complaints were significantly higher in the tanners than the controls subjects (χ^2^ test). A small percentage of the workers had other health problems like body pain, general weakness, diabetes, hypertension, arthritis, goiter, bronchospasm, conjunctivitis and frequent fever. While some of the workers had more than one health problem, a number of tannery workers reported no symptoms (28%). There were very few cases of hypertension, diabetes and allergic diseases in the control group.

### Types of tannery work

The tannery workers enrolled in this study came from the wet blue section (working moist Cr (III) oxide-tanned blue leather, 21%), drum section (washing unfinished leather, 17%), technical section (machine operators that shave, spot remove and split leather, and move unfinished leather from one section to another etc., 11%), and chemical section (lime and other chemical treatments of raw hides, color spray etc, 19%). Some workers were involved with removing hair from hides, cutting, washing, drying and finishing (20%). Additionally, some workers involved in unloading, storing and weighing chemicals (8%), and packing (4%) were included in this study because of their close contacts with possible exposure.

### Serum chromium levels

Thirty serum samples of the tannery workers involved in different types of work (at least 3 from each job category, results shown in Table 2), who had been employed in the tanneries for 9 to 30 years were used for the determination of serum Cr levels. Their mean duration of work exposure was 22.4 ± 4.0 years. The Cr content in these samples varied from 0.67 to 107.0 μg/dL, with a mean value of 26.97 ± 21.11 μg/dL. Workers involved in cleaning chemical-treated leather, chemical storing, mixing, soaking and raw stacking had the highest level of Cr in serum (44.2 μg/dL) compared to those working in the finishing sections (15.3 μg/dL). A total of 30 serum samples from randomly selected unexposed control subjects were analyzed for Cr and the results varied from 0.4 to 18.3 μg/dL, with a mean value of 7.38 ± 6.81 μg/dL. Statistical analysis revealed that these long-term exposed tannery workers (N=30) had significantly higher Cr concentrations in serum than the control subjects (p<0.05, Mann-Whitney U test).

**Table 2:** Levels of Chromium in Serum of Tannery Workers Across Job Types

Tannery worker jobs	Number of tanners	Cr (μg/dL)
Cleaning treated leather, chemical storing, mixing, soaking and raw stacking	4	44.2 ± 16.0
Chemical treatment of raw hide	4	23.3 ± 10.8
Wet blue and drum sections, lime addition	4	22.7 ± 2.0
Wet blue finishing, spraying chemicals	3	26.0 ± 19.6
Leather splitting operators	6	25.3 ± 16.2
Leather shaving	6	28.9 ± 39.7
Vacuum drying of finished leather, packing of finished goods, working in glue section	3	15.3 ± 5.5

All values are expressed in Mean ± SD.

### Serum immunoglobulin G and A levels

The mean levels of serum IgG and IgA in the tannery workers, 11.67 ± 3.58 g/L and 1.50 ± 0.62 g/L, respectively, were significantly lower than the corresponding values in the control subjects (*Table 3*).

**Table 3:** Serum IgG, IgA, IgE, Complements C3 and C4, and Bactericidal Activity in Tannery Workers and Controls

Parameters tested	Reference value	Tannery workers N=135	Control subjects N=100	Statistics^#^ (p-value)
IgG (g/L)	7.0–16.0^21^	11.67 ± 3.58	13.66 ± 2.65	<0.05
IgA (g/L)	0.7–4.0^21^	1.50 ± 0.62	1.92 ± 0.66	<0.01
IgE (IU/mL)	0–200^@^^21^	340 ± 275	153 ± 265	<0.01
C3 (g/L)	0.9–2.1^21^	0.78 ± 0.22	1.01 ± 0.23	<0.05
C4 (g/L)	0.1–0.4^21^	0.19 ± 0.07	0.23 ± 0.08	<0.05
Bactericidal activity (%)^*^	up to 100^20^	89.7 ± 3.22	93.2 ± 2.14	<0.001

^#^Student's t-test;

^*^Results of 35 randomly picked samples from each group.

^@^Values in Western countries. All values expressed in Mean ± SD.

### Serum immunoglobulin E levels

In the present study, the serum IgE level of the control subjects varied from 3–750 IU/mL, and the level varied from 4–950 IU/mL in the tannery workers. Statistical analysis showed that the IgE level in the tannery workers was significantly higher (p< 0.01) than in the control subjects (*Table 3*).

### Serum levels of complement components

About 40% of the tannery workers had a serum complement C3 level within the normal range (normal value: 0.9 - 2.1 g/L), whereas 60% were below the normal level. The mean levels of serum complements C3 and C4 in the tannery workers were significantly lower than those in the control subjects (*Table 3*).

### Assessment of complement function

**Figure 2:**
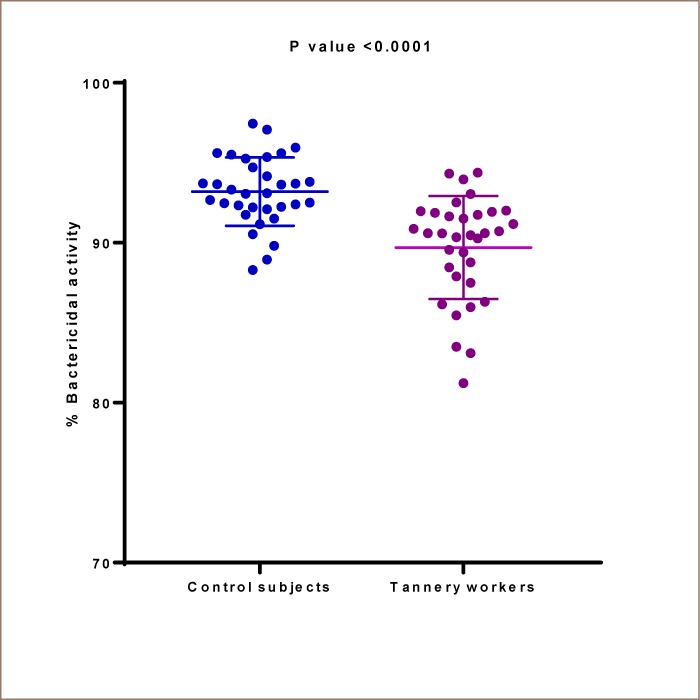
Bactericidal activity in tannery workers and control subjects Scatter plot of the percentage of Escherichia coli DH5α cells killed (bactericidal activity) by serum complements from the control subjects and tannery workers. Complements from the control subject killed a mean value of 93.2 ± 2.1% bacterial cells, while those from the tannery workers killed 89.7 ± 3.2% of bacterial cells (p<0.001).

A total of 35 control subjects and 35 tannery workers were investigated for the bactericidal activity of serum complements. Since complements are heat-labile and readily activated to generate split products, these samples were thawed only once. Thus, samples used in other assays could not be tested for this function. The number of colonies grown in PBS (negative controls) varied from 600 - 2420 × 10^6^ colony forming units (cfu)/mL with a mean value of 1437 × 10^6^ cfu/mL. Upon treatment with serum complements, the number of colonies formed varied from 26 - 242 × 10^6^ cfu/mL, with a mean value of 110 × 10^6^ cfu/mL for the control subjects, while the number of colonies varied from 60 - 324 × 10^6^ cfu/mL with a mean value of 133 × 10^6^ cfu/mL for the tannery workers. Compared to PBS, complements of both the tannery workers and control subjects exhibited significant bactericidal activities (p<0.001 for both). The bactericidal activity of serum complements was calculated in terms of percentage of killing. It was found that complements from the control subjects killed 88.3 to 97.1% of bacterial cells with a mean value of 93.2 ± 2.1%. On the other hand, complements from the tannery workers killed 81.2 to 94.4% of bacterial cells with a mean value of 89.7 ± 3.2%, which was significantly lower (p< 0.001) than for the control subjects (*Table 3 and Figure 2*).

### Data correlation analyses

Correlation analyses of the immunological, nutritional and Cr data were carried out to investigate the relationships between different parameters in the study subjects. These findings are shown in Table 4. There was a significant inverse correlation between the levels of serum IgG with the duration of occupational exposure of the tannery workers, suggesting suppression of secondary humoral immune response. Bactericidal activity also showed an inverse correlation with duration of exposure. There were significant negative correlations between the levels of serum IgE and age of the tannery workers and control subjects, showing coherence of these analyses. Immunoglobulin G, IgA and C3 were all inversely associated with serum Cr, while C4 showed inverse, weak or no relation, and bactericidal activity had no relation with Cr. Serum IgE had a significant inverse correlation with BMI of the tannery workers. There were no significant correlations between the levels of serum IgG and C3 with the BMI of tannery workers. Additionally, the BMI of the control subjects showed no relationship with the respective levels of IgE, IgG and C3 (data not shown).

**Table 4:** Correlation of Immunology Data, Chromium, Age and BMI of Tannery Workers

Immunology parameters	Correlation coefficients with			
	Chromium in serum (μg/dL)	Duration of Cr exposure (years)	Age (years)	BMI (kg/m^2^)
Serum IgG (g/L)	−0.111	−0.330^*^	−0.280^*^	0.109
Serum IgE (IU/mL)	0.051	−0.215	−0.246^*,a^	−0.336^**^
Serum IgA (g/L)	−0.129	−0.072	−0.090	0.114
Complement C3 (g/L)	−0.142	0.027	−0.103	0.083
Complement C4 (g/L)	−0.042	0.114	−0.074	0.065
Bactericidal activity (%)	0.022	−0.203	0.346	0.215

^#^Spearman rho correlations;

^*^p<0.05;

^**^p<0.001;

^a^ A significant negative correlation was also found between the levels of serum IgE and age of the control subjects (r =−0.452, p< 0.01).

There was a significant linear correlation between the levels of serum IgE and blood eosinophils in the control subjects (r = 0.578, p < 0.001) which was not found in the tannery workers. Eosinophils were counted from blood films stained with Giemsa stain using an Olympus microscope.

There was a significant linear correlation between the levels of serum IgE and blood eosinophils in the control subjects (r = 0.578, p < 0.001) which was not found in the tannery workers. Eosinophils were counted from blood films stained with Giemsa stain using an Olympus microscope.

## Discussion

The present study examined serum immunoglobulins, complement proteins and immune function in long-term Cr-exposed tannery workers and the findings were compared with unexposed control subjects. During leather manufacture, the widely used Cr(III) is oxidized to form Cr(VI), which is more soluble, highly toxic and capable of penetrating skin with its ability to form free radicals, and thus can damage the skin of tannery workers.^22^ One study found that tannery workers in Sialkot, Pakistan exhibited hematological, hepatic and renal function impairment which was attributed to enhanced oxidative stress and inflammatory changes in the body.^23^ Tannery workers in Kanpur, India showed high morbidity (about 40%) possibly due to Cr exposure and respiratory illness.^24^

Tannery effluents have also been found to suppress immune function in tilapia.^25^ A previous study reported that the tannery wastewaters of Hazaribagh have extreme values of chemical oxygen demand and very high concentrations of Cr.^26^ Chromium mainly enters the body through inhalation and ingestion, and a small amount can enter through direct skin contact. Chromium is highly toxic by dermal and inhalation routes and can cause lung cancer, nasal ulcers, contact hypersensitivity and dermatitis.^27^ One study on Cr-related health effects in Kenya found significantly higher respiratory and dermatological complaints among tannery workers compared to an unexposed control group.^22^ The present study found that 45.1% of tannery workers had contact dermatitis, exhibited by rough and itchy skin and rashes and 12.3% had respiratory problems (*Table 1*). Both complaints were significantly higher than the unexposed controls, which may have been caused by direct contact and inhalation of various chemicals, including Cr. It was observed that many tannery workers in Hazaribagh were not using gloves, masks and protective boots while handling various chemicals, despite policies requiring protective equipment. This warrants further study.

We found decolorized skin on the upper and lower limbs of 16.4% of the tannery workers exposed to chemicals (*Table 1*). The purposes of the chemicals used in the tanning processes are to remove hair from hides, harden, decolorize, soften, lime and de-lime hides, among other functions. As the tanners sometimes perform this work with bare hands, these chemicals may produce similar results on their own skin, i.e., decolorized skin, caused hardening, roughness and skin rash. Chromium and other chemicals are well known to cause inflammation. Chromium exposure through inhalation leads to respiratory complications like breathing and chest problems, wheezing, cough etc., which could be an influencing factor for elevated levels of serum immunoglobulin E. Immunoglobulin E plays an important role in the onset of allergic responses, although IgE has a beneficial role against parasitic worms. In Western countries, the normal serum concentration of IgE is 0–200 IU/mL, whereas in tropical countries this level may vary up to 2000 IU/mL.^21^ A previous study suggested that bronchial reactivity can develop after exposure to fumes from metal plating with Cr and nickel, and an IgE response may be seen even in previously non-allergic individuals.^28^ Studies have reported a prevalence of various allergic diseases among tannery workers in Bangladesh, and serum IgE levels were significantly higher in the occupationally-exposed tannery workers.^6^,^18^

The effect of Cr on the immune system has been investigated in several in vitro studies. Borella et al. studied the effect of Cr (III) and Cr (VI) on phytohaemagglutinin-induced blastogenesis in human lymphocytes.^11^ Cr (VI) has shown stimulatory effects at nanomolar concentrations and inhibitory effects at micromolar concentrations, while Cr (III) has no effect. Injection of cobalt-chromium particles into the peritoneal cavity of mice showed significantly higher levels of cobalt and Cr in the blood at 5 weeks after injection, while the release of IL-2 and IL-4, proliferation of both T and B cells, and antibody production by lipopolysaccharide-stimulated B cells were all significantly inhibited after 3 weeks of injection. These observations suggest that metal-induced immunosuppression may play an important role in the development of infection in patients with implanted prostheses.^13^ A study by Vasant et al. stated that human lymphocytes cultures die in the presence of both Cr(V) and Cr(VI) as these salts elicited toxic effects on the cells, and the mode of cell death appeared to be apoptosis due to the formation of reactive oxygen species.^29^ A recent study suggested the toxic effects of Cr(VI) on blood leukocytes could be due to mitochondrial injury and DNA damage of the cells leading to carcinogenicity.^30^

The present study found significantly elevated levels of serum Cr in chronically exposed tannery workers (26.97 μg/dL) compared to the control subjects who also had high levels of Cr in serum, 7.38 μg/dL (the normal value of Cr has been suggested to be 2–3 μg/dL for an unexposed population).^31^ Table 2 further shows tannery workers directly involved in cleaning chemical-treated leather, chemical storing, mixing etc. had as high as 44.2 μg/dL Cr compared to those working in the finishing and packing sections, 15.3 μg/dL. These results suggest tannery workers have an alarmingly high risk of Cr toxicity, while other residents of Dhaka city are also exposed to Cr, possibly through consumption of contaminated food or inhalation of leather dusts and fumes emanating from the nearby tanneries. However, owing to increased concern over the health effects of Cr exposure and environmental pollution caused by tannery wastes in Dhaka city, most of the tanneries in Hazaribagh have been relocated to the newly built leather industrial estate in Savar following a High Court order.

In the present study, serum IgG and IgA levels of the tannery workers were found to be inversely associated with the level of Cr in serum (*Table 4*). Various factors such as Cr(V) and Cr(VI)-induced apoptosis of lymphocytes^29^, infection and route of exposure may decrease IgG levels. Immunoglobulin A is believed to be the first line of defense in the respiratory tract. The secretion of IgA bathes the mucosal area of the respiratory system to prevent infection and invasion of microbes.^32^

The toxic effect of chronic exposure might reduce free IgA in the lining of the respiratory tract. There could be other mechanisms for decreased production of this protein, including polyclonal activation of B cells by bacterial lipopolysaccharide that switches towards IgE production with a concomitant suppression of other isotypes of immunoglobulins, IgG and IgA. Lower levels of IgA could increase the possibility of various nasal or air-borne infections, possibly caused by fungus and other opportunistic pathogens like *Candida albicans*. We found that about 14% of the tanners had fungal and bacterial infections on body surfaces, which was in agreement with a previous report that found tanners to be prone to fungal and other types of infection.^33^

Complement C3 is the major protein in the complement system that can kill bacteria directly by making pores on the target cell surface. Complement C3 becomes activated via activation pathways during infection. In the present study, both complements C3 and C4 levels were found to be significantly lower in the tannery workers, suggesting impaired function of the classical complement pathway. The bactericidal activity of the workers was significantly lower, which could be due to lower levels of C3 and C4. Correlation analyses of the data showed bactericidal activity had no relation with Cr, which could be due to the small number of samples analyzed for this function. However, bactericidal activity was inversely correlated with the duration of work exposure. The lower levels of IgG and IgA presented in this study are in agreement with the observation of Qian et al., suggesting that adverse changes in the immunological status in tannery workers are possibly caused by occupational exposure to chromate and other chemicals.^34^ The authors further reported that levels of interferon gamma, IgG and IgA were all inversely associated with whole blood Cr, while C3 and C4 were positively associated with whole blood Cr. Our findings partially support those of Qian et al., as IgG and IgA showed an inverse correlation with serum Cr (evenly distributed between blood cells and plasma), however, C4 showed an inverse (weak or no) relation, while C3 was inversely associated with Cr (*Table 4*).^34^

The present study found serum IgE levels to be significantly elevated in the tannery workers. It should be noted that IgE is only produced in response to a select group of antigens (allergens and intestinal parasites). We found a significant linear correlation of IgE with eosinophil count in the control subjects which was not found in the tanners (*Table 4*), suggesting elevated IgE levels were not due to intestinal helminths, rather it could be a direct effect of inhalation of Cr-containing leather dusts and fumes of chemicals used in tanneries. In the present study, 12.3% of tannery workers were found to be suffering from respiratory illness. A similar study reported that respiratory diseases (16.7%) were mainly responsible for a higher morbidity among exposed tannery workers in Kanpur, India.^24^ It is possible that workers with respiratory diseases may ultimately develop bronchial obstruction leading to lung cancer, which is quite common among Cr-exposed tannery workers.^1^,^27^ It is important to note that no other study, except one previous work with a small sample size, found elevated serum IgE levels in tannery workers and thus cannot be compared with other results.^18^

The present study showed a significant inverse correlation of IgE with BMI among tannery workers (*Table 4*), suggesting that underweight/malnourished workers were more affected by the toxic effects of Cr that induced allergic responses leading to respiratory diseases; elevated IgE levels could be due to the toxic effects of Cr. Immunoglobulin G and IgE both had an inverse correlation with duration of work exposure, although unlike IgG, the effect on IgE was not significant. Immunoglobulin G and IgA are important components of humoral immunity and thus decreases in levels generally indicates a decline in the immune system, although elevated IgE signals a deteriorating health status. The present study found that 15% of tannery workers were underweight (BMI <18.5), which was significantly higher (p<0.001) than the control population. The inverse correlation of IgE with BMI suggests that the poor nutritional status of the workers may be associated with Cr-induced allergic responses leading to respiratory diseases and elevated IgE in the tannery workers.

## Conclusions

Workers in the leather tanning industry in Hazaribagh face a serious threat of Cr toxicity. Tannery workers directly exposed to Cr salts had considerably higher levels of Cr in serum than those working in the finishing sections. The present study found significantly lower levels of serum IgG, IgA, C3 and C4, but significantly higher levels of IgE in workers with, skin problems, infections, allergies and respiratory illness. Tannery workers also had lower bactericidal activity, suggesting impaired immune function. Levels of IgG, IgA and C3 were all inversely correlated with serum Cr. Direct exposures to Cr and prolonged work duration were correlated with adverse health effects among these workers. Elevated levels of serum IgE could be a toxic effect of Cr on the tannery workers.

Tannery workers in Hazaribagh were observed working with bare hands and feet. The use of gloves, rubber boots and gas masks could greatly reduce Cr exposure in these workers. Monitoring occupational conditions, assessment of leather dust, Cr particles, fumes of organic solvents, and fungal and bacterial spores in the tannery air could reduce health risk factors for these workers. Additionally, regularly scheduled physical health examinations would benefit the health and longevity of the tannery workers.

### Study limitations

The present study was subject to certain limitations. The sample size was inadequate and might not reflect the full picture of this population. It must be noted that only 30 samples of chronically exposed tannery workers and 30 samples of control subjects were investigated for Cr contents due to a shortage of serum samples. Another limitation of the study was exclusion of female tannery workers. This was because very few female tanners worked in sections using Cr for 2 years or more. Additionally, bactericidal activity was assessed on 35 random samples from each group as there was a scarcity of unthawed serum samples with which to study complement function. Therefore, the results might not reflect the whole population; furthermore, correlations of some parameters might have been missed. Finally, the study could not take into account other toxic chemicals used in tanneries, which might have affected the results.
