# Supplementary material for: Serum Immunoglobulin Levels and Complement Function of Tannery Workers in Bangladesh
Source: J Health Pollut. 2019 Mar 14;9(21):190308. doi: 10.5696/2156-9614-9.21.190308 (PMC6421957; doi:10.5696/2156-9614-9.21.190308)
Supplement: Supplementary file 1 [file hapn-9-21-190308_s01.docx]

**Supplemental Material**

**QUESTIONNAIRE FOR SAMPLE COLLECTION FROM THE TANNERY WORKERS**

Entry No. Date:

**A. General information**

1. Name of the participant:

2. Address of work place:

3. Age (yrs): Sex: Male/Female Education: Primary/Secondary/Higher

4. No. of family members:

5. Monthly income (Tk.):

6. Duration of work (yrs): 7. Working hours (hrs/day):

8. Type of work:

9. Smoking habit: Yes/No If yes, for how long? Years:

10. Height (cm): Weight (kg): Waist (cm): Hip (cm):

**B. Medical information**

11. Blood pressure: mmHg

12. Pulse rate:

13. Examination of upper limb: Rough: Skin color change: Itch: Rash: Palm:

14. Examination of lower limb: Rough: Skin color change: Itch: Rash: Foot:

15. Chest examination: Clear: Noisy: Breathing problem: Cough: Asthma:

16. Eye examination: Normal: Irritation: Anemic:

17. Tongue examination: Normal: Inflamed: Other:

18. Eating disorder (if any): None: Anorexia: Other (specify):

19. Physical health Weakness: Abdominal pain: Headache: Nausea: body pain: Other

information: (specify):

20. Blood group: O A B AB +ve -ve
